# H3N2 Virus as Causative Agent of ARDS Requiring Extracorporeal Membrane Oxygenation Support

**DOI:** 10.1155/2014/560208

**Published:** 2014-01-09

**Authors:** Adriano Peris, Giovanni Zagli, Pasquale Bernardo, Massimo Bonacchi, Morena Cozzolino, Lucia Perretta, Alberta Azzi, Giovanni Cianchi

**Affiliations:** ^1^Anaesthesia and Intensive Care Unit of Emergency Department, Careggi Teaching Hospital, Florence, Italy; ^2^Department of Heart and Vessels, Careggi Teaching Hospital, Florence, Italy; ^3^Department of Clinical and Experimental Medicine, University of Florence, Florence, Italy

## Abstract

Pandemic influenza virus A(H1N1) 2009 was associated with a higher risk of viral pneumonia in comparison with seasonal influenza viruses. The influenza season 2011-2012 was characterized by the prevalent circulation of influenza A(H3N2) viruses. Whereas most H3N2 patients experienced mild, self-limited influenza-like illness, some patients were at increased risk for influenza complications because of age or underlying medical conditions. Cases presented were patients admitted to the Intensive Care Unit (ICU) of ECMO referral center (Careggi Teaching Hospital, Florence, Italy). Despite extracorporeal membrane oxygenation treatment (ECMO), one patient with H3N2-induced ARDS did not survive. Our experience suggests that viral aetiology is becoming more important and hospitals should be able to perform a fast differential diagnosis between bacterial and viral aetiology.

## 1. Introduction

Influenza viruses represent an important cause of severe lower respiratory disease. Pandemic influenza virus A(H1N1) 2009 was associated with a higher risk of viral pneumonia in comparison with seasonal influenza viruses [1, 2]. During the pandemic and the following influenza epidemic season 2010-2011, this led to an increased frequency of hospitalization in Intensive Care Units (ICUs) and of Acute Respiratory Distress Syndrome (ARDS) that is the most severe type of acute lung injury. The influenza season 2011-2012 was characterized by the prevalent circulation of influenza A(H3N2) viruses. Whereas most H3N2 patients experienced mild, self-limited influenza-like illness, some patients were at increased risk for influenza complications because of age or underlying medical conditions.

Here we show our experience of treating two cases of adult patient with ARDS due to H3N2 pneumonia treated with venovenous extracorporeal membrane oxygenation (ECMO).

## 2. Methods

### 2.1. Setting

The patients were admitted to the Intensive Care Unit (ICU) of Careggi Teaching Hospital (Florence, Italy) as ECMO Referral Center in season 2011-2012. Data were collected from ICU databases and the Italian Group for the Evaluation of Interventions in Intensive Care Medicine database (GiViTI Margherita Project, Istituto Mario Negri, Bergamo, Italy).  Table 1 summarized baseline and clinical characteristic of patients. Internal Review Board approved the study and informed consent for data publication was obtained.

### 2.2. Ventilation Strategies and Extracorporeal Membrane Oxygenation (ECMO)

ECMO was used to support the respiratory function after the failure of standard protective ventilation strategy (6 mL/kg tidal volume normalized on ideal body weight and plateau pressure lower than 30 cm H_2_O). The positive end-expiratory pressure (PEEP) applied was fixed at lower flex in the pressure/volume curve [3]. ECMO treatments were venovenous (Maquet Rotaflow Centrifugal Pumps with Quadrox-D oxygenators, Maquet, Rastatt, Germany) and biocoated circuits were used. Avalon double lumen cannulas (Avalon Elite Bi-Caval Dual Lumen Catheter 27-31 Fr., Avalon Laboratories, Rancho Dominguez, CA, USA) were inserted through internal right jugular vein. Cannulas position was confirmed by transesophageal echocardiography [3].

### 2.3. Laboratory Tests

Influenza virus identification and typing was performed by RT-real time PCR, using specific set of primers and specific TaqMan probes (following the indications of the Italian National Influenza Centre, National Health Institute, Rome). The primers and the probes targeted, respectively, a sequence of M gene specific for influenza type A viruses, of M gene specific for type B influenza, and different sequences of HA gene, each specific for the seasonal H3 and H1 subtype. In addition, for the detection of the pandemic H1 2009 virus, the set of primers and probe proposed by the Centers for Disease Control and Prevention were used [4]. The RT-real time PCR for the M gene of influenza type A viruses was employed as a quantitative assay to assess the load of virus shedding, using serial dilution of an RNA standard prepared in our laboratory. This calibrator consisted of the RNA sequence transcribed by the T7 RNA polymerase on the template of the product of the real time amplification of the M gene, cloned in the pGEM-T Easy Vector (Promega).

## 3. Cases Presentation

### 3.1. Patient 1

M. G., a 66-year-old man in treatment with immunosuppressive drugs due to rheumatologic diseases, experienced cough and sneezing in the beginning without other symptoms. He was not vaccinated against influenza. Clinical conditions had been worsening for a few days, so that the patient was admitted to a local hospital for respiratory distress after 19 days from initial symptoms. At Emergency Department (ED) admission, he was hypoxic, with clinical and radiological signs of ARDS and he was immediately transferred to the ICU. After 6 days our ECMO center was contacted; patient was then evaluated on-site by the mobile ECMO-team and ECMO treatment was started in the peripheral hospital. The patient was transferred to Careggi Hospital in Florence.

Diagnostic imaging showed an improvement of consolidation areas of upper pulmonary lobes and an increase of interstitial lung disease without pleural effusion. Echocardiography showed mild pulmonary hypertension. PCR assays made on pharyngeal swab and bronchoalveolar lavage were positive for viral genomes of H3N2 virus (10^5^ copies/mL). Bronchoalveolar lavage resulted negative for bacterial aetiology. Oseltamivir (10 mg twice) was initiated. From day 8 onwards, lung functions and imaging improved and ECMO weaning was started until ECMO-off (zero gas flow) for 12 hours. On day 11, ECMO system was removed.

After ECMO removal, the patient remained on supportive mechanical ventilation and he was cooperative and able to perform respiratory physiotherapy. As consequence of pulmonary inflammation, patient developed progressive pulmonary fibrosis and pulmonary hypertension. Moreover, PCR for CMV was found positive (11014 copies), and therapy with ganciclovir was started. Five days after ECMO removal, the patient showed a septic exacerbation with fever up to 39°C, increased laboratory markers of infection, and worsened gas exchange which needed an increase in ventilator support. The following day, the patient's respiratory and, consequently, hemodynamic status deteriorated despite increasing ventilator setting. A high dosage of inotropes was required. Microbiological examination showed a positive PCR for HSV1. The treatment with specific antiviral drugs was unsuccessful and the patient developed multiple organ failures and after 18 days of post-ECMO removal the patient died.

### 3.2. Patient 2

F. M., a 76-years-old man, experienced fever (as high as 40°), cough, and dyspnoea for 3 days. He was not vaccinated against influenza and the patient was referred to ED because of persisting of respiratory symptoms. The CT-scan performed before admission showed extensive consolidations mostly at the superior right lobe and both inferior lobes. In both throat swab and bronchoalveolar lavage PCR for influenza virus A(H3N2) was positive (10^5^ copies/mL), so oseltamivir was initiated. Bronchoalveolar lavage resulted negative for bacterial aetiology. For the worsening of gas exchange and patient's respiratory fatigue, clinicians proceeded to intubation and mechanical ventilation. For the persistent severe hypoxemia despite high PEEP levels, after three days the decision to start venovenous ECMO was made.

During ECMO treatment the patient was kept mildly sedated and he was ventilated with lung protective mode. No major complication occurred during ECMO treatment. Respiratory exchange improved and weaning from ECMO was started and ended after 8 days when ECMO system was removed. After various spontaneous breathing trials, he was able to maintain spontaneous breathing and he was discharged after 23 days of ICU stay.

## 4. Discussion

In pandemic 2009 influenza A(H1N1), many patients developed critical illness and needed to be treated with ECMO [5]. In our experience during pandemic 2009 influenza A(H1N1) [6] we observed a low mortality rate in H1N1-induced ARDS patients, as also reported in larger series of H1N1 patients [7–9], if compared to bacterial ARDS. This difference can be attributed mainly to the fact that H1N1 respiratory failure presents a relatively benign course when adequately treated, if compared to non-H1N1-induced ARDS, even when ECMO was necessary.

During the influenza epidemic season 2011-2012, we had two cases of severe respiratory infection caused by seasonal influenza A viruses, subtype H3N2, slightly different from the vaccine virus A/Perth/16/2009, belonging to the clade A/Victoria/208/2009. In these patients, the H3N2 virus induced severe inflammation and epithelial necrosis of bronchi and bronchioles with extension into the alveoli causing diffuse alveolar damage. This process leads to ARDS. This observation strengthens what our experience, following the H1N1 pandemic [10], strongly suggests that the screening for influenza virus should be performed systematically in ICU patients during seasonal influenza epidemics, in particular from November to March [11].

## 5. Conclusions

Viral aetiology of acute respiratory failure is becoming more relevant, especially in young and old compromised people. Our cases show influenza A(H3N2) viruses as one of the factors causing pneumonia associated to significant morbidity and mortality.

## Figures and Tables

**Table 1:** Clinical characteristic of H3N2-pneumonia patients.

Patient	Age (years)	Gender	Body mass index	Comorbidity	PaO_2_ (mmHg)/FIO ratio at admission	Oxygenation index: mean airway pressure (cmH_2_O) ∗ FIO_2_ ∗ 100/PaO_2_ (mmHg)	Mechanical ventilation duration (days)	ECMO support	ICU length of stay	Outcome
M. G.	66	Male	24	Rheumatoid arthritis, Sjogren Syndrome	107	15	30	Yes	30	Nonsurvived

F. M.	76	Male	26	Diabetes Mellitus, arteriopathy	80	6	20	Yes	23	Survived
